# Clinicopathological profile of subgemmal neurogenous plaques: A 52-year retrospective study of 22 cases

**DOI:** 10.4317/medoral.27233

**Published:** 2025-08-16

**Authors:** Julliany Taverny Sousa, Hannah Gil de Farias Morais, Débora Frota Colares, Éricka Janine Dantas da Silveira, Roseana de Almeida Freitas, Lélia Maria Guedes Queiroz, Lélia Batista de Souza

**Affiliations:** 1 Department of Oral Pathology, Federal University of Rio Grande do Norte, Natal, RN Brazil; 2 Federal University of Rio Grande do Norte. Multicampi School of Medical Sciences, Caicó, Brazil

## Abstract

**Background:**

This study describes the clinical and histopathological profile of 22 subgemmal neurogenous plaques (SNP) through a 52-year retrospective study conducted at an Oral Pathology referral center in the Northeast of Brazil.

**Material and Methods:**

Clinical data (age, biological sex, symptoms, clinical presentation, lesion size, duration at diagnosis, growth rate, implantation, and consistency) were retrieved from biopsy records. Hematoxylin and eosin-stained slides from all selected cases were re-evaluated.

**Results:**

SNPs accounted for 0.12% (n = 22) of all lesions diagnosed at the center. A predominance among female patients was observed (n = 20; 90.9%), with most diagnoses occurring in the fifth to sixth decades of life (mean 57.5 ​ ± 12.19 years). Clinically, the lesions typically presented as slow-growing but painful, reddish papules. Five cases were associated with oral lymphoepithelial cysts. Histologically, common features included spindle cells, subepithelial nerve plexuses, ganglion cells, mast cells, lymphoid tissue, and germinal centers.

**Conclusions:**

These findings underscore the importance of recognizing the clinical and histopathological characteristics of SNPs to avoid misdiagnosis as neural tumors.

** Key words:**Subgemmal nerve plexus, subgemmmal neural plaque, taste buds, tongue.

## Introduction

Subgemmal neurogenous plaque (SNP) is a neural structure located in the tongue, commonly associated with taste buds (1). It was first described as a tortuous neural proliferation within subepithelial nerve plexuses and ganglion cells, exhibiting neurofibroma-like features, and referred to as “subepithelial nerve plexus” (2). The currently preferred designation, “subgemmal neurogenous plaque”, more accurately conveys both its anatomic location and the tissue of origin without implying a pathological lesion (3,4).

The etiology of SNP remains debated in literature. Initially, it was thought to represent a reactive neuronal dysplasia, the result of trophic influences from gustatory nerve fibers, or a manifestation of small-fiber sensory neuropathy (5). Subsequent work has proposed that SNPs are in fact normal neural anatomical structures forming a morphological and functional complex with taste buds (6).

To date, several investigations have detailed clinical and morphological characteristics of SNPs, noting that their non-specific clinical appearance of this condition often leads to misdiagnosis as reactive or neoplastic soft-tissue lesions. Histopathologically, SNPs may closely resemble neural neoplasms, further complicating accurate diagnosis (4,7). Thus, to improve understanding of the clinical presentation and diagnostic criteria for SNPs, this study conducted a clinical-pathological analysis of 22 cases diagnosed as SNPs. The analysis focused on their diverse clinical presentations, as well as their morphological and immunohistochemical characteristics, with the goal of increasing the awareness among clinicians and oral pathologists regarding this structure as a potential differential diagnosis for oral tongue lesions.

## Material and Methods

- Study design

This retrospective, cross-sectional and descriptive study was conducted on all cases previously diagnosed as SNPs over a 52-year period (January 1970 - December 2022) archived at an oral Pathology referral center. All histopathological diagnoses of SNP were initially provided by a certified oral pathologist and subsequently re-examined during the study by two oral pathologists (JTS, HGFM) for the morphological characterization described below. Additionally, cases originally diagnosed as oral lymphoepithelial cysts (OLCs) located at the lateral border of the tongue were reassessed, due to the well-documented association between this lesion and SNPs in the literature (4).

Clinical data, including age, sex, skin color, symptomatology, lesion size (mm), time to diagnosis (months), clinical presentation, lesion color, growth type, consistency, implantation, and clinical diagnosis, were retrieved from biopsy records stored at the service.

Five-micrometers-thick sections were obtained and subjected to routine hematoxylin-eosin (H&E) staining, followed by analysis under a light microscope (Five-Head Microscope, Nikon Eclipse-E200, Tokyo, Japan). Histopathological analysis evaluated the following morphological parameters: the presence of taste buds; the presence and distribution of neural bundles; connective tissue characteristics; the intensity and localization of inflammatory infiltrate, as well as its relationship with neural bundles and lymphoid tissue; the presence of lymphoid aggregates and germinal centers; the occurrence of mast cells and ganglion cells; vascular distribution; and pseudoepitheliomatous hyperplasia (8). For a more precise characterization of the cellular components present in SNPs, immunohistochemical reactions were performed using antibodies against S-100 (dilution 1:200; Dako), neuron-specific enolase (NSE) (dilution 1:800, Dako) and cytokeratin-7 (CK7) (dilution 1:200, Dako). These reactions were conducted on 3-µm histological sections mounted on glass slides coated with organosilane adhesive (3-aminopropyltriethoxysilane; Sigma Chemical Co., St. Louis, MO, USA), following a standardized protocol.

- Statistical analysis

Data were analyzed using the IBM Statistical Package for the Social Sciences (v22.0; IBM Corp., Armonk, USA) and descriptive analysis was performed on the frequencies of clinical and histopathological data.

## Results

- Demographic characteristics

Among all oral and maxillofacial lesions diagnosed at the referral service, 22 (0.12%) were SNPs. Clinical data revealed a predominance of white, female patients (female-to-male ratio 10:1) (Table 1). Most cases were diagnosed during the fifth and sixth decade of life, with a mean age of 57.5 ​​ ± 12.19 years (range 36-77 years).

- Clinical findings

**Figure 1:**
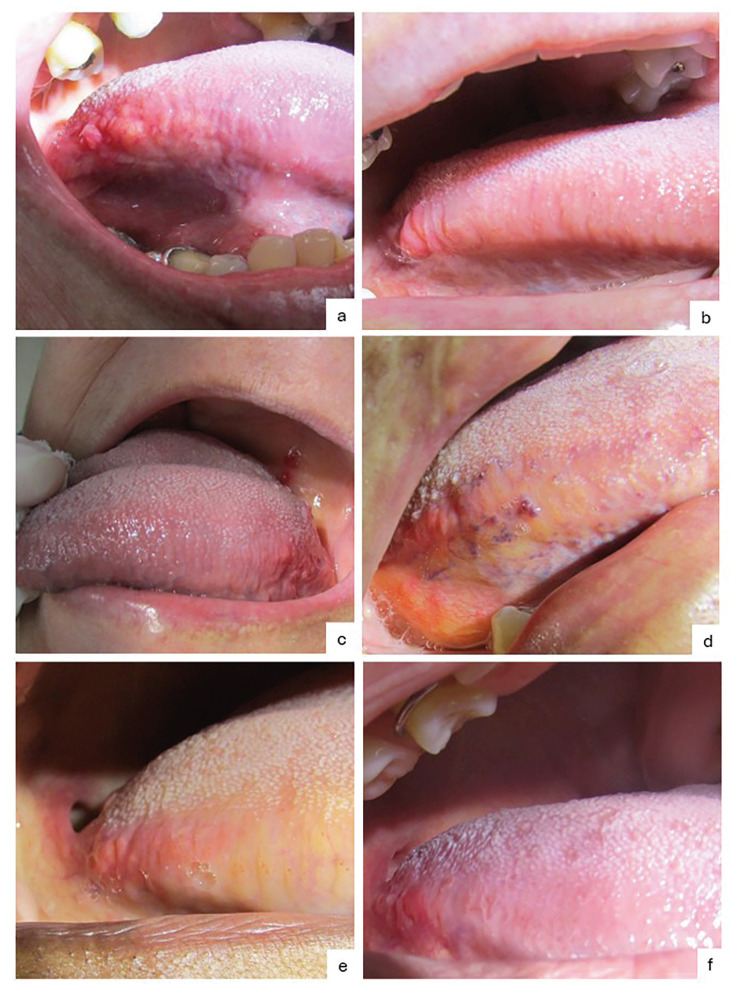
Clinical aspects of subgemmal neurogenous plaque observed in this study. a) Symptomatic reddish nodular mass on the lateral border of the tongue in a 63-year-old female patient. b) Symptopmatic reddish smooth macule on the lateral border of the tongue in a 53-year-old female patient. c) Symptomatic reddish papule near the ventral tongue in a 69-year-old female patient. d) Asymptomatic reddish papule adjacent to tongue varicosities in a 62-year-old female patient. e) Symptomatic yellow mass on the lateral border of the tongue in in a 67-year-old female patient. f) Symptomatic slight reddish swelling on the lateral border of the tongue in a 40-year-old female patient.

Subgemmal neurogenous plaques typically presented as slow-growing, painful, reddish papules or nodules with a soft consistency (Fig. 1). The lesions had a mean diameter of 5 mm (range 1-20mm), and an average duration prior to diagnosis of 4 months (range 0-120 months). Bilateral occurrences were uncommon (n ​​= 5; 22.7%) (Table 1).

Surgical excision was the primary treatment modality (n = 17; 77.3%). In most instances, histopathological diagnoses aligned with the clinical hypotheses documented in the biopsy reports (n = 11; 50.0%). Five cases initially diagnosed as oral lymphoepithelial cyst were found to exhibit SNP features upon histological review, and were included in this study. Additional clinical features are summarized in Table 1.

- Microscopic findings

Table 2 provides an overview of the main histopathological characteristics observed. All SNPs exhibited proliferation of spindle cells (n = 22; 100%) frequently interspersed with taste buds (n = 18; 81.8%). Subepithelial neural bundles were typically organized within the fibrous connective tissue, which exhibited predominantly mild vascularization. Ganglion cells and mast cells were identified in approximately 45% and 77% of the sample, respectively.

**Figure 2:**
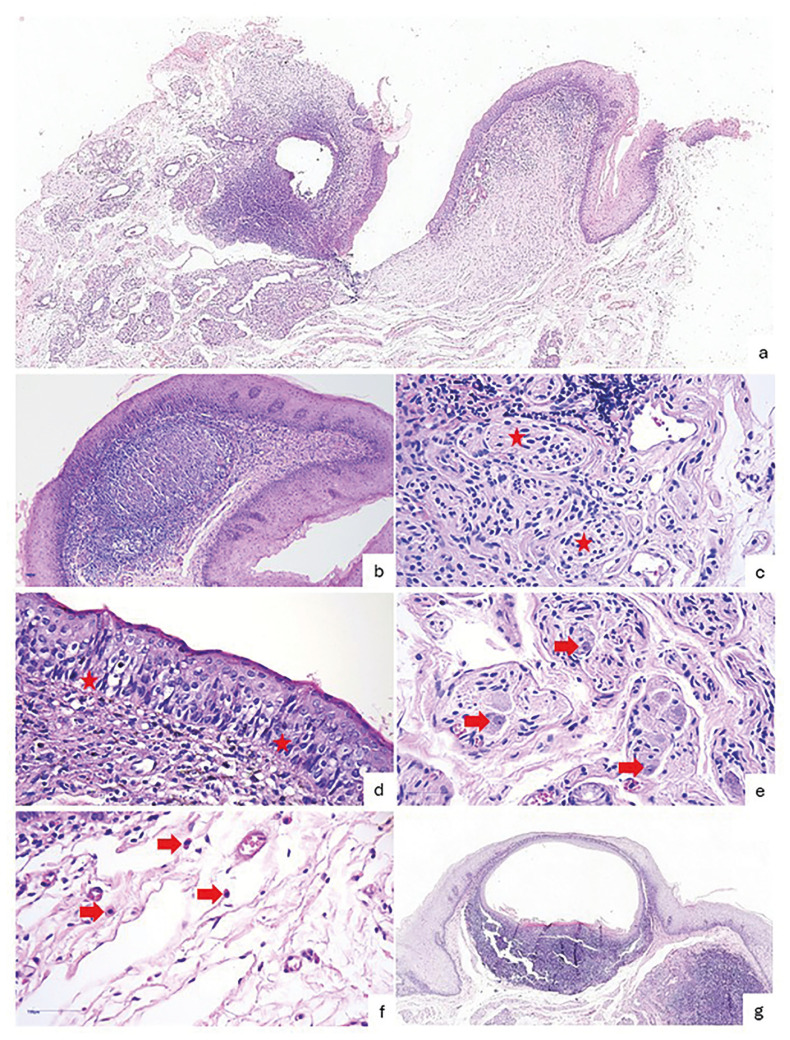
Histopathological features of subgemmal neurogenous plaques (SNPs). a) SNP exhibiting proliferation of fusiform cells beneath the epithelial lining (upper right). An associated oral lymphoepithelial cyst is also observed (upper left) (H&E, 40x). b) Germinal center within lymphoid tissue located beneath the epithelial lining (H&E, 200x). c) Lamina propria showing proliferation of fusiform neural cells, organized as irregular neural bundles enveloped by a delicate perineurium (red stars) (H&E, 400x). d) Epithelial lining of the oral mucosa containing taste buds (red stars) (H&E, 400x). e) Ganglion cells within irregular neural bundles (red arrows) (H&E, 400x). f) Mast cells were frequently present near neural proliferation (red arrows) (H&E, 400x). g) Oral lymphoepithelial cyst associated with a SNP (H&E, 40x).

All cases displayed a predominantly moderate, mononuclear inflammatory infiltrate. More than half of SNPs contained lymphoid tissue (n = 18; 81.8%) with germinal centers (n = 13; 59.1%), and three cases (13.6%) showed pseudoepitheliomatous hyperplasia. Histopathological features are illustrated in Fig. 2.

**Figure 3:**
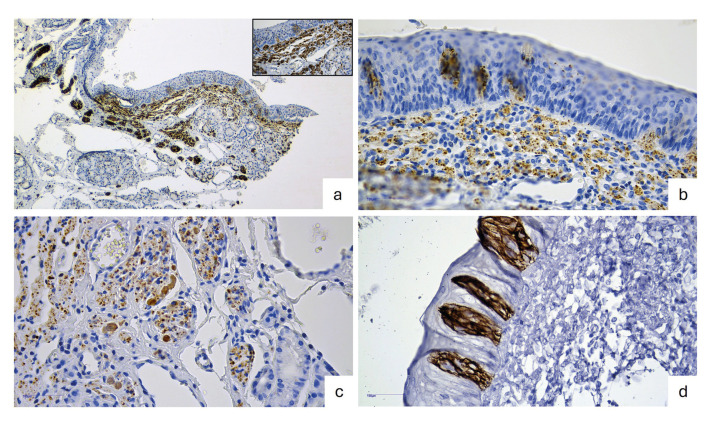
Immunohistochemistry of subgemmal neurogenous plaques (SNPs). a) S-100-immunoreactive neural cells in the spindle cell component of SNP (IHC, ×100). S-100 positivity is also observed in superficial cells (inset, IHC, original magnification ×400). b) Spindle cells in the superficial component showing positivity for neuron-specific enolase (NSE) (IHC, ×400). c) NSE also highlights mature ganglion cells in the deeper portion (IHC, ×400). d) Intense CK7 staining in taste bud (IHC, ×400).

Immunohistochemical findings of SNPs are illustrated in Fig. 3. Spindle cells located in the superficial zone revealed positivity for S-100 and NSE. In the deeper zone, ganglion cells also exhibited NSE immunoreactivity. Additionally, CK7 expression was observed in taste buds located within epithelial areas adjacent to SNPs.

## Discussion

This study examined the clinical, histopathological, and additional relevant criteria for the management of SNPs through a retrospective analysis of 22 cases diagnosed at an Oral Pathology referral center located in Brazil’s Northeast region. SNPs are characterized by a biphasic nerve organization (2). When symptomatic, their diagnosis poses a challenge for clinicians and less-experienced pathologists because their clinical presentations and histopathological features overlap with those of other hyperplasic and neoplastic lesions of the tongue (6,9).

The results demonstrated that SNPs comprised less than 1% of all cases diagnosed at the center over a 52-year period. These lesions were more prevalent in adult females in their fifth decade of life, consistent with previous investigations (4,7,10). However, the true prevalence of SNPs in the general population may be higher, since these lesions are typically asymptomatic and thus rarely reported by patients. Consequently, they may not attract clinical attention. This hypothesis is supported by the high prevalence of SNPs observed in an autopsy series (8). In practice, excision is usually reserved for cases in which patients present symptoms or when the lesion’s appearance raises suspicion of other conditions, such as squamous cell carcinoma (6).

Clinically, SNPs presented as slow-growing erythematous papules or nodules, in line with earlier descriptions (4,7). In the present study, most patients reported a burning sensation that justified surgical excision. Although SNPs represent a normal anatomical structure, the mechanism underlying their pain remains poorly understood. It has been hypothesized that chronic trauma to enlarged SNPs symptomatology may directly stimulate nerve fibers or do so indirectly via inflammatory mediators (6,11).

Subgemmal neurogenous plaques exhibit low prevalence and may mimic other red or yellow oral lesions, thereby posing a diagnostic challenge (6,12). In this study, clinical diagnoses ranged from SNPs to various hyperplastic and erythroplastic lesions. The differential diagnosis of SNPs includes transient lingual papillitis, oral lymphoepithelial cyst (OLC), reactive lesions, benign neural neoplasms, granular cell tumor, leukoplakia, and erythroplakia (7,11).

A noTable observation was the frequent co-occurrence of SNPs and OLCs on the posterior lateral border of the tongue - five of the 22 cases exhibited this association (9,13,14). Given their shared location, this synchronous presentation likely reflects coincidence rather than a direct pathological relationship.

Microscopically, SNPs are characterized by a biphasic subepithelial nerve plexus of small, deep nerve fascicles and taste buds embedded within the epithelium (2). This organization was a common finding in our series. Typically, SNPs present a superficial zone - parallel to the epithelial surface -, and a deeper zone. The superficial zone contains spindle, wavy cells dispersed within the lamina propria or arranged in twisted cords against a collagenous background, resembling a neurofibroma-like pattern. The deeper zone comprises irregular, tortuous neural bundles ensheathed by delicate perineurium (6,15). In this study, the deeper bundles were more often demonstrated an organized arrangement.

The histopathological findings of SNPs, namely spindle cell proliferation and irregular nerve bundles, overlap significantly with those of benign neural neoplasms, including neurofibroma or traumatic neuroma, complicating diagnosis for pathologists unfamiliar with this structure. A review of head-and-neck neural neoplasm cases revealed that only 30% of cases initially classified as neural neoplasms were confirmed as such upon re-evaluation, while most of the remainder, particularly tongue lesions, were reclassified as SNPs (1). These findings align with previous studies on this topic (2,8,15).

Additional histopathological criteria important for the diagnosing SNP include lymphoid aggregates with germinal centers, pseudoepitheliomatous hyperplasia, and conspicuous populations of ganglion cells and mast cells (6,8,15,16). In this study, most cases exhibited these features alongside fusiform cell proliferation.

Immunohistochemical analysis in this study was consistent with previous reports (6,7). Immunoreactivity for S-100 and NSE confirmed the neural origin of SNPs, with both markers demonstrating positivity in spindle cells of the superficial zone and within neural bundles, thereby illustrating the characteristic biphasic pattern. Moreover, NSE expression was detected in mature ganglion cells in the deeper zone. Finally, CK7 immunostaining demonstrated numerous taste buds in epithelial areas adjacent to regions of neural cell proliferation.

Although SNPs are recognized as normal anatomical structures at the posterolateral border of the tongue, their development remains unclear. It has been proposed that SNPs represent either a form of reactive neural dysplasia or native neural structures associated with taste buds that may become symptomatic (2,10). Because SNPs are benign, surgical intervention is generally unnecessary except in symptomatic cases, in which biopsy is warranted to exclude reactive lesions or soft-tissue neoplasms (6).

This study’s strengths include a comprehensive demographic, clinical, and histopathological characterization of SNPs. To the best of our knowledge, this series represents one of the largest cohorts of SNPs reported to date, making it a valuable reference for clinicians and oral pathologists when formulating differential diagnoses. Nonetheless, this study has some limitations, including the absence of certain clinical data and the consequent inability to control confounding variables, as well as inherent difficulties in establishing causal relationships. Furthermore, as a single-center study, it is susceptible to selection bias during data collection. Collectivelly, these factors may limit the generalizability of our findings. These limitations are to be expected, particularly given the long-term, retrospective nature of the data analyzed in this study.

In summary, our findings indicate that SNPs are uncommon anatomical structures that predominantly affect the posterolateral border of the tongue in females in their fifth decade of life. Clinically, these lesions present as small, slow-growing nodules or papules that may be painful. Accurate recognition of SNPs by clinicians and oral pathologists is essential for precise diagnosis and appropriate management.

## Figures and Tables

**Table 1:** Absolute and relative distribution of cases of neurogenous subgemmal plaque according to clinical parameters.

Parameters		n (%)
Sex	Male	2 (9.1)
	Female	20 (90.9)
Age (years)	30-39	1 (4.5)
	40-49	5 (22.7)
	50-59	7 (31.8)
	60-69	7 (31.8)
	70-79	2 (9.5)
Skin color	White	6 (27.3)
	Brown	8 (36.3)
	Black	4 (18.2)
	NR	4 (18.2)
Pain	Present	11(50.0)
	Absent	9(40.9)
	NR	2 (9.1)
Clinical presentation	Nodule	4 (18.2)
	Papule	8 (36.4)
	Ulcer	1 (4.5)
	Patch	3 (13.6)
	Plaque	1 (4.5)
	NR	5 (22.7)
Color of the lesion	Red	11 (50.0)
	White	3 (13.6)
	Yellow	4 (18.2)
	Similar to mucosa	4 (18.2)
Rapid growing	Yes	3 (13.6)
	No	10 (45.5)
	NR	9 (40.9)
Consistency	Softened	10 (45.5)
	Fibrous	6 (27.3)
	Similar to mucosa	3 (13.6)
	NR	3 (13.6)
Clinical diagnosis	Neurogenous Subgemmal Plaque	10 (47.6)
	Oral lymphoepithelial cyst	3 (14.3)
	Papillitis	2 (9.1)
	Other	6 (27.3)
	NR	1 (4.5)

Abbreviations: NR, not reported.

**Table 2:** Absolute and relative distribution of cases of neurogenous subgemmal plaque according to morphological parameters.

Parameters		n (%)
Taste buds	Present	18 (81.8)
	Absent	4 (18.2)
Neural bundles	Regular	17 (77.3)
	Irregular	4 (18.2)
	Absent	1 (4.5)
Connective tissue	Fibrous	12 (54.5)
	Loosen	8 (36.4)
	Variable	2 (9.1)
Inflammatory infiltrate	Discrete	7 (31.8)
	Moderate	9 (40.9)
	Intense	6 (27.3)
Location of inflammatory infiltrate	Next to neural bundles	8 (36.4)
	Next to lymphoid tissue	2 (9.1)
	Next to neural bundles and lymphoid tissue	11 (50.0)
	Distant to neural bundles and lymphoid tissue	1 (4.5)
Lymphoid tissue	Present	18 (81.8)
	Absent	4 (18.2)
Germinal centers	Present	13 (59.1)
	Absent	9 (40.9)
Mastocytes	Present	13 (77.3)
	Absent	5 (22.7)
Ganglion cells	Present	10 (45.5)
	Absent	12 (54.5)
Pseudoepitheliomatous hyperplasia	Present	3 (13.6)
	Absent	19 (86.3)
Vascularization	Discrete	11 (50.0)
	Moderate	5 (22.7)
	Intense	6 (27.3)
